# Assessment of the Influence of the Geometrical Shape of the Damper on the Efficiency of an Ultrasonic Operation Piezoelectric Transducer

**DOI:** 10.3390/s23249662

**Published:** 2023-12-06

**Authors:** M. S. Vechera, S. I. Konovalov, R. S. Konovalov, B. I, V. M. Tsaplev, A. D. Soloveva, J. Lee

**Affiliations:** 1 LLC “Constanta US”, Ogorodny Lane 21, Saint Petersburg 198097, Russia; vms@constanta.ru; 2 Department of Electroacoustics and Ultrasonic Engineering, Saint Petersburg Electrotechnical University “LETI”, Prof. Popova 5, Saint Petersburg 197022, Russia; rskonovalov@etu.ru (R.S.K.); valery@convergences-fr.ru (V.M.T.); adsoloveva@stud.etu.ru (A.D.S.); 3 Department of Smart Manufacturing, Changwon National University, Changwon National University Road 20, Changwon 51140, Republic of Korea; ee.boris@changwon.ac.kr; 4 School of Mechanical Engineering, Changwon National University, Changwon National University Road 20, Changwon 51140, Republic of Korea; jaesun@changwon.ac.kr

**Keywords:** nondestructive testing, piezoelectric transducer, damper

## Abstract

The results of a study on the geometric shape of the damper on the efficiency of the ultrasonic piezoelectric transducer are presented. In particular, a damper in the form of a truncated cone is considered, the generatrix of which has an inclination angle α relative to the diameter of the piezoceramic plate. The shape of the damper in the form of a truncated cone is chosen based on the a priori assumption that this helps to increase the path of the wave in the damper material due to numerous reflections in it. A criterion for the efficiency of damper operation is proposed. The optimal (from the point of view of the damper efficiency) value of the angle α was determined theoretically and experimentally. The technology of its production is described. Satisfactory agreement between the results of theoretical and experimental studies was noted.

## 1. Introduction

The damper is one of the most critical structural elements of an ultrasonic piezoelectric transducer (PET). It is designed to increase the transmitter bandwidth and reduce the duration of transient processes. The mechanical damping of the active element of the probe is most often used to achieve this goal, and it is a universal means for suppressing the inertial properties of PETs [1,2,3,4,5,6,7,8,9,10,11,12,13,14]. Mechanical damping is currently used in the manufacture of single ultrasonic piezoelectric transducers with a large aperture (NDT, biomedical diagnostics). The damper introduces active losses into the oscillatory system. The presence of a reactive component disrupts the efficiency of its operation. A purely active input acoustic impedance of the damper is achieved only when the influence of ultrasonic waves reflected from its rear side is excluded. This implies a requirement for the damper material, which must be with a large ultrasonic attenuation coefficient. This is important because the damper has very limited dimensions. In addition, the damper material must have a high specific acoustic impedance, ideally the same as piezoceramics. The back side of the damper often has scatterers, from which the waves are reflected in different directions. As a result, the length of their path inside the material significantly increases. This leads to greater signal attenuation and does not allow reflected waves to hit the piezoelectric element directly after reflection from the rear end.

An analysis of the publications shows that dampers with powder fillers are currently the most widely used. Here, a fine powder of a material with a high specific acoustic impedance (e.g., tungsten) is introduced into the matrix (epoxy resin, sealant, polyurethane). The papers [9,10] present specific materials used in such damper designs, the relationships between components, etc. The wide distribution of these types of dampers was probably facilitated by the well-established manufacturing technology, which achieved a high degree of identity of the properties of the transducers. In addition, the manufacturing technology of this type of damper does not require significant time or labor costs, which is an essential circumstance in the mass production of probes.

According to [10], the operation of a damper of this type becomes most effective when the size of the absorber particles is comparable with the wavelength, especially with composite fillers. Thus, in addition to powder filler with high specific acoustic impedance, to ensure the scattering of ultrasonic waves, a material with sharply different acoustic impedance value, e.g., crumb rubber, can also be introduced into the damper material. The end part of the damper can be manufactured, for example, of a spherical shape, with the center offset relative to the damper axis, which helps to increase scattering and eliminates the influence of reflected waves. In [10], profiled type dampers are also presented, having different shapes of the end surface, as well as dampers of complex shape, combining, for example, a horn part and a ball with a recess in the upper part. Typically, such dampers are made of metals with a specific acoustic impedance close to that of piezoceramics. From the point of view of the authors of this paper, the disadvantage of probes with such dampers is their ability to work only with plates of a specific diameter effectively. For plates with different diameters, it is necessary to select the shape of the cone generatrix again.

One can mention the damper [15] of the ultrasound transducer, the manufacturing of which consists of placing the piezoplate into a cast form, which is then filled with melted damping material with filler and then cooled until curing. The disadvantage of this method is the inability to obtain a damper with a characteristic impedance close to the acoustic impedance of piezoelement. This is because increasing the percentage of the filler to increase the impedance of the damper leads to the growth of the viscosity and heterogeneity of the entire mass. This complicates its filling and gluing to the piezoelement. The insufficient attenuation of ultrasound waves in such dampers leads to the necessity of increasing the size of the entire transducer. In addition, it causes some doubts by repeating the parameters of the PET equipped with a damper of this type. A similar drawback can be attributed to the damper described in [16]. The method of its manufacturing is that on the back of the fat–fat and prolonged in a modified resin, heated above the boiling temperature of the water, the piezoelement is poured into the filler to a height of at least half-waves at the resonant frequency of the transducer, poured with a binder and then cured.

The influence of this deficiency on the properties of the damper is significantly reduced in its design, presented in [17], which describes a two-layer damper, which is performed in two stages. First, a tiny sublayer of the damping composition is applied to the surface of the piezoelement (epoxy resin with fine tungsten powder). Further, after polymerization, the mentioned sublayer is poured with a layer of polyurethane with tungsten powder. After that, the transducer is centrifuged, which ultimately allows one to achieve a smooth decrease in the damper impedance as it moves from the surface of the piezoelement. This helps to reduce the influence of reflections from the back of the damper. However, the presence of the second layer entails the increase in its height.

In [18], a method of manufacturing a damper is described, which consists of the following. A layer of damping material is applied to the surface of a piezoelectric element fixed on a piezoelement. The specific impedance of the damping material is close to the impedance of the piezoelement. Next, the damping material is heated to the melting point. The layer of melt hardens on the surface of the piezoelement; a damper is formed without intermediate gluing layers. Then, the temperature is reduced, and the powder of the same damping material is introduced into the form and pressed. Due to the gradual decrease in the melt temperature, part of the powder melts, part is compressed, and a damper is formed with a heterogeneous macrostructure in height with a high ultrasound attenuation. The disadvantage of the described damper is the difficulty of obtaining the identity of the PET parameters.

Some other technologies of damper manufacturing [17,19] are known. These technologies increase the damping properties of the damper because a damping mass is placed on a vibrator or centrifuge and subjected to vibration processing that helps the compaction of the damping mass. As a result, the heavy particles of the filler are concentrated near the area of the damper that adjoins the piezoelement.

The analysis of the publications allows us to argue that, when developing dampers, the major efforts of the authors have been aimed at studying their specific technological features. We have studied the influence of the form of the damper on the effectiveness of its operation to a much lesser extent. For example, it is known that dampers often have the form of a truncated cone; however, the issues related to determining the optimal geometric parameters of these types of dampers remain unexplored. In this paper, we discuss some issues related to this problem.

## 2. The Setting of the Problem

**Figure 1:**
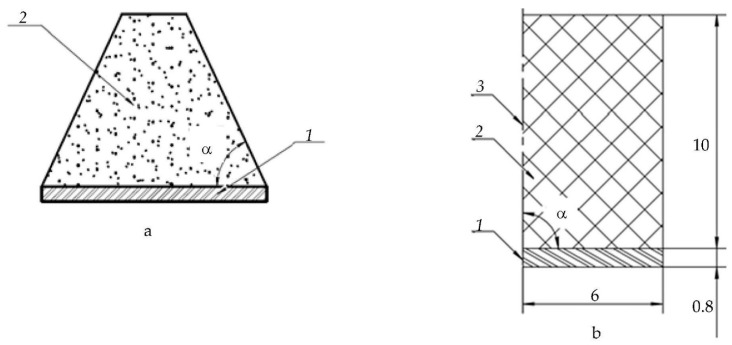
The setting of the problem: (**a**) sketch; (**b**) the geometry of the transducer; *1*—round piezoplate, *2*—damper, *3*—symmetry axis.

The setting of the problem can be formulated as follows. Figure 1a shows a piezoelectric plate, the back surface of which is in contact with the damper. The following designations are used in the figure: *1*—piezoplate; *2*—a damper that has the form of a truncated cone. The resulting cone has an angle of inclination α regarding the line corresponding to the diameter of the plate. The shape of the damper in the form of a truncated cone is selected based on the a priori assumption that this helps to increase the path of the wave inside the damper due to its numerous pores. It is obvious that the effectiveness of the damper depends on the value of the angle α. The goal is to determine the optimal value of α, in which the signal falling on plate 1 will be minimal due to the reflections inside the damper.

The solution to the problem was carried out in two stages (first, theoretically, by simulating a numerical experiment using the COMSOL Multiphysics 6.1 software, and second, subsequent comparison of the results at each of the stages).

For both the theoretical and experimental studies, PZT ceramics (Russian TsTS-19) was chosen as the material for the piezoplate. Its properties are described in [20]. The material of the damper is a mixture of the modified epoxy resin of KDA with the hardener of the ETAL-45M and the filler. As a filler, a fine powder of the PV-1 tungsten was used with an average particle size of 0.8…1.7 μm, made according to TU 14-22-143-2000 (1:1 by weight, and the mass fraction of the resin is indicated taking into account the hardener).

The parameters of the damper, which are necessary for the calculation, were determined experimentally. We studied these using a precision meter of the distribution rate and attenuation of the longitudinal and transverse waves of the production of SSRIE “Acoustics”, which uses PET based on an active element from the lithium niobate, having resonant frequencies 1.25 and 2.5 MHz. The emitting–receiving system is axisymmetric, and the experimental setup consisted of a pulse generator and an oscilloscope. The attenuation was determined by measuring the ratio of pulse signal amplitudes on both sides of the plane-parallel damper specimen. The velocities of the longitudinal and transverse waves were determined through measuring the time of the pulse signal traveling through the specimen.

The result of this experiment for measuring the damper parameters is shown in Table 1.

While performing the theoretical and experimental research, the plate was excited by a signal in the form of a single-period meander with amplitude $V_{\mathrm{in}}=200$ V and duration $\tau =1/f_{0}$, where $f_{0}$—the resonant frequency of piezoplate.

## 3. Numerical Modeling

Figure 1b shows the initial geometry adopted for the theoretical solution of the problem using the COMSOL Multiphysics 6.1. The figure shows the plate’s geometric dimensions and the damper’s height. The geometry of the problem under consideration is axisymmetric, which should be noted. As a result, the figure shows only the half of the model. It is worth noting that a truncated cone with an angle $\alpha =90^{\circ }$ transforms into a cylinder. This version of the damper is shown in the figure.

To simulate the operation of the PET, Solid Mechanics, Electrostatic, and the multiphysics Piezoelectric Effect Modules were used to describe the propagation of elastic waves. To describe an electric circuit connected to a piezoelement, an Electrical Circuit Module was used. The size of the mesh was selected from the Courant–Friedrichs–Lewy criterion [21], which establishes the dependence between the spatial Δ*x* and time steps Δ*t* and the velocities of the longitudinal $c_{l}$ or transverse $c_{t}$ waves:

(1) $$\mathrm{CFL}=\frac{\Delta t}{\Delta x}c_{l,t}.$$

In this model, the spatial step is equal to one-twelfth of the wavelength at the central frequency, the coefficient CFL=0.1, and the transverse wave velocity is chosen as a wave velocity, since at the same frequency the length of the transverse wave is less than that of the longitudinal one. The mesh consisted of 7800 elements (changing depending on the angle) and 74,396 degrees of freedom, and the time step from (1) is 7 μs. The angle α during the calculation varied from 60° to 90° with step 2°.

To evaluate the effectiveness of the damper, the following criterion was used: exciting the plate with an electric signal of a given amplitude and duration causes an acoustic signal to emit into the damper. The same plate receives the reflected signal. The ratio of the amplitudes of these two signals for the various values of the angle α gives the desired dependence.

**Figure 2:**
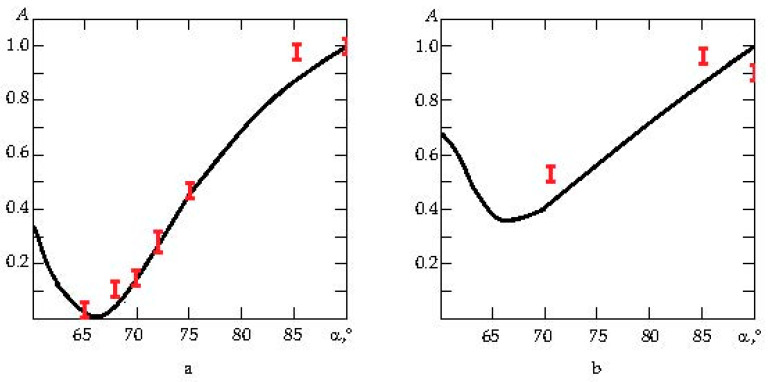
(**a**) Change in the coefficient *A* as the function of the angle of inclination α for the excitation frequency 2.5 MHz; (**b**) change in the coefficient *A* as the function of the angle of inclination α for the excitation frequency 1.25 MHz.

The result of the numerical simulation is presented in Figure 2. It shows the ratio of the amplitudes of electrical voltages at the surface of the piezoelement: the voltage from the reflected wave $V_{\mathrm{out}}$ to the applied to the piezoplate $V_{\mathrm{in}}$, depending on the angle α of inclination of the side wall of the damper (forming a truncated cone). The abscissa axis is for the inclination in the generatrix in degrees, and the ordinate axis is for the ratio $A=V_{\mathrm{out}}/V_{\mathrm{in}}$. The data in the figure indicate that the best result at α = 64° ± 66° at the frequency 2.5 MHz can be achieved. Indeed, at this value of the angle α, the parameter *A* reaches the value 0.0001. For comparison, one can cite data from the same Figure 2a: if $\alpha =60^{\circ }$ and $\alpha =70^{\circ }$, then the values of *A* are 0.33 and 0.14, respectively. At the frequency 1.25 MHz (Figure 2b), the optimal value will be $\alpha =66^{\circ }$, and A=0.36.

The analysis of the data given in Figure 2 allows us to argue that with α = 64° ± 66°, one can obtain the best result to achieve the minimum amplitudes of the reflected signals.

**Figure 3:**
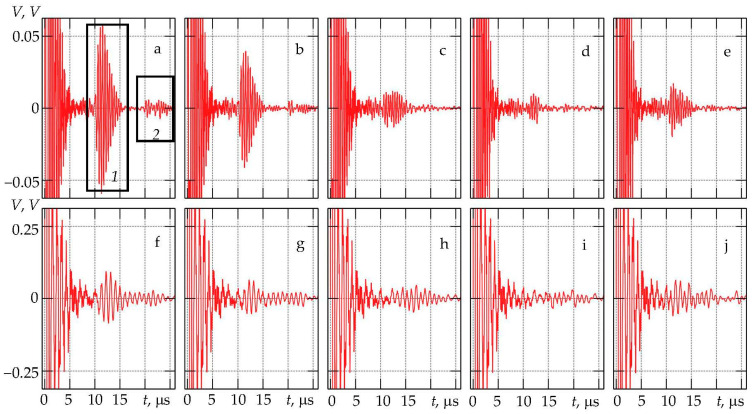
Piezoelement output voltage at 1.25 MHz at the angles of inclination α: (**a**) 60°, (**b**) 62°, (**c**) 64°, (**d**) 66°, and (**e**) 68° and at 2.5 MHz at the angles of inclination of α: (**f**) 60°, (**g**) 62°, (**h**) 64°, (**i**) 66°, and (**j**) 68°. *1*—signal reflected from the back side of the damper, *2*—signal caused by re-reflections inside the damper.

For a more detailed study of the processes in the damper, we investigated the output electrical voltage of the piezoelement, depending on the time. Figure 3 presents the change in the electrical voltage *V* at the output of the piezoplate over time at various values of the angle of inclination of the forming truncated cone α.

If the dynamics of changes in the amplitudes of the reflected signals at the output of the piezoplate, as a function of the angle α at a frequency of 2.5 MHz (Figure 3f–j), is considered, the existence of a certain value of the angle α, at which the damper works most efficiently can be noted. Within the interval α = 60° ± 66° the signals *1* and *2* practically disappear when α = 64° ± 66°. The level of multiplication of the reflected signals also reduced practically to zero. The further growth of α within the range 66° ± 90° leads to an increase in the signal amplitude due to the multiple overlapping of pulses inside the damper. Similar processes are observed at 1.25 MHz (Figure 3a–e). The signal amplitudes are higher than at 2.5 MHz, although the noise level is higher, which may indicate a weaker attenuation of the multiplied reflected and transformed waves in the damper.

The increase in the signal amplitude caused by multiple reflections inside the damper at α < 64° ± 66° can be explained, because the back reflecting wall of the damper begins to “function as a reflective plane”. Indeed, the analysis of the signal fronts inside the damper showed that, at α < 64° ± 66°, the signal is first reflected from the back of the damper and does not change due to multiple reflections from the generatrix.

**Figure 4:**
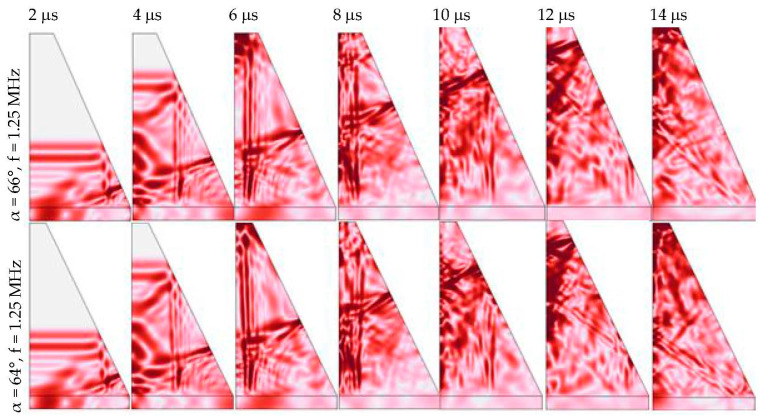
Changes in the signal front over time at 1.25 MHz for α = 64° and α = 66°.

As an example, Figure 4 shows changes in the wavefront in time for the frequency 1.25 MHz for α = 64° and α = 66°. One can note that near the back of the damper, the signal receives multiple reflections.

## 4. Experimental Research

For experimental verification of the calculated values, the piezoceramics TsTS-19 with 2.5 and 1.25 MHz resonance frequencies and 12 mm diameter are used. To minimize the errors caused by the spread of the parameters of the piezoelectric elements, all the piezoelements were selected in groups in terms of the resonance frequency and capacity. The spread of the resonance frequencies during the experiment did not exceed ± 10 kHz of the average value for the group, and the capacity did not exceed 30% of the average.

**Figure 5:**
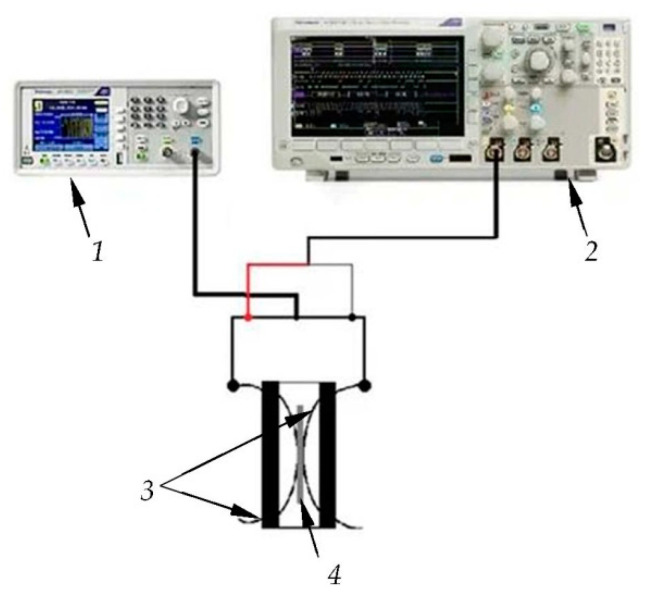
The experimental setup to determine the resonant frequencies of piezoplates: *1*—signal generator; *2*—oscilloscope; *3*—clamping electrodes; *4*—piezoplate.

Before use, the piezoplates were selected to obtain the same resonance frequency. To achieve this, a signal generator AFG1022 (TEKTRONIX, Beaverton, OR, USA) and an oscilloscope MDO3012 (TEKTRONIX, Beaverton, OR, USA) were used. Figure 5 shows the experimental setup. The generator applied the continuous sinusoidal signal of 10 V to the electrodes of the piezoplate clamped in the equipment. The frequency step of the exciting signal was 1 kHz. The change in the signal amplitude was monitored using an oscilloscope. The minimum amplitude of the signal on the oscilloscope corresponded to the resonance frequency. The frequency deviation of each plate was no more than 10 kHz.

We manufactured the specimens using 3D printing technology from water-soluble PVA plastic. The equipment consisted of an injection mold in the form of a truncated cone with a hole in top for filling the damping mass. The base of the form was a circle with a radius equal to the radius of piezoplate. Such manufacturing technology is rather cheap, easy, and quick with regard to preparing forms, with a lack of technological maps, the possibility of automatic printing, and no requirements for the consistency of control. In addition, using a 3D printer, one can create a shape of any size and configuration. To fix the piezoceramics and forms, double-sided foamed tape was used.

The damping composition was prepared according to the previously described technology (a mixture of the modified epoxy resin of KDA with the hardener of the ETAL-45M and the filler), and after degassing, it was poured into molds. The filling was carried out using a syringe without a needle. After 48 h, the forms with a polymerized damper were removed from the adhesive tape and placed into water to dissolve the mold. It took about 3–4 h to soften and partially dissolve the mold. The rest of the mold, which did not dissolve during this time, was removed mechanically.

**Figure 6:**
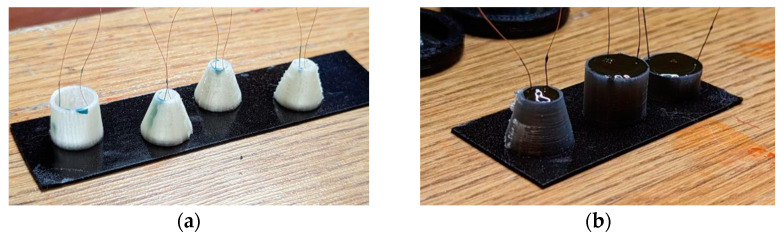
Photos: (**a**) piezoplates with injection molds for various angles α; (**b**) piezoplates with dampers.

According to the described technology, 30 samples were made using piezoplates with the frequency of 2.5 MHz and 9 samples with 1.25 MHz. Figure 6a shows photos of the piezoplates with injection molds, and Figure 6b shows the piezoplates with dampers.

Specimens having the slope of the cone generatrix 90°, 85°, 75°, 72°, 70°, 68°, and 66° were made using the 2.5 MHz plates—three specimens for each value of the angle of inclination, which was made to prevent random errors in manufacturing.

Specimens with 1.25 MHz piezoplates with an inclination of cone generatrix 90°, 85°, and 70° were also made in triplicate.

All these prepared experimental PETs were studied in the same way, as under the theoretical investigations. They were excited by a signal in the form of a single-period meander at the frequency of the piezoplate resonance. The signal amplitude was 200 V, generated by the ultrasonic flaw detector UCD-50. Signals from the back free wall of the damper came from the digital output of the flaw detector and were recorded using UDOscill v2.3.0.4 software. Figure 2 shows how changes in the ratio of the maximum amplitude of the signal were reflected from the back of the damper to the amplitude of the probing signal, depending on the angle of inclination (see red marks). The red marks indicate the range of values A for a particular angle of inclination α obtained experimentally. This allows one to compare the theoretical and experimental data. Excellent agreement between the simulated and experimental data is observed at the resonant frequency of 2.5 MHz. The points corresponding to the experimental data differ from the calculated not more than about 8% (see Figure 2a). When 1.25 MHz piezoplates were used, the mismatch rose to 15%.

## 5. Conclusions

The paper describes the experiment and the simulation of the influence of the damper’s geometric form on the effectiveness of its work. The following conclusions can be formulated:

1. The criterion for assessing the effectiveness of the damper in the form of the ratio of amplitudes of signals reflected from the free end of the damper and the probing impulse was proposed. This criterion is convenient for the operational control of PET properties during their production.
2. The FEM model of the PET with a conical damper was developed and experimentally confirmed.
3. The technology for the damper manufacturing with water-soluble mold was developed and tested.

The effectiveness of a conical damper with the tungsten powder filler mixed with the resin of KDA in a ratio of 1:1 turned out to be higher in comparison with the damper of a cylindrical shape. The damper, made from exactly such a mixture, can be effectively used to manufacture transducers with a frequency not less than 2.5 MHz. For lower frequencies, selecting another damping composition with other fillers or its other ratio is necessary. The maximum efficiency of the conical damper is achieved at the inclination of the cone generatrix is within α = 64°…66°.

To further advances in this area, it would be beneficial to focus on the following topics:

- Evaluation of the effectiveness of dampers with more complex shapes (for example, having a recess on the back side);
- Estimation of the amplitude and duration of the output acoustic signal depending on the shape of the damper.

## Figures and Tables

**Table 1:** Damper parameters.

$\mathrm{Longitudinal}\;\mathrm{Wave}\;\mathrm{Velocity}\;c_{l}$, m/s	$\mathrm{Transverse}\;\mathrm{Wave}\;\mathrm{Velocity}\;c_{t}$, m/s	Density ρ, kg/m^3^	Attenuation of Longitudinal Waves $\delta_{l}$, dB/mm	Attenuation of Transverse Waves $\delta_{t}$, dB/mm
2083 ± 36	950 ± 25	2218 ± 45	at frequency 1.25 MHz 0.7 ± 0.05	at frequency 1.25 MHz 1.0 ± 0.06
			at frequency 2.5 MHz 0.75 ± 0.03	at frequency 2.5 MHz 2.5 ± 0.08

## Data Availability

Data are contained within the article.
